# Anévrismes intracrâniens multiples

**DOI:** 10.11604/pamj.2016.23.36.7834

**Published:** 2016-02-09

**Authors:** Rachid Ammor, Assou Ajja

**Affiliations:** 1 Neurosurgery, Military Hospital My Ismail, Meknes, Morocco

**Keywords:** Hémorragie méningée, artériographie cérébrale, anévrismes multiples, Subarachnoid hemorrhage, cerebral arteriography, multiple aneurysms

## Image en medicine

Il s'agit d'une jeune femme de 33 ans, suivie pour HTA sous monothérapie depuis un an, admise aux urgences pour des céphalées d'installation brutale. L'examen clinique trouve une patiente consciente, TA=13/7, avec présence d'un syndrome méningé franc sans fièvre. La TDM cérébrale a montré une hémorragie méningée au niveau de la vallée sylvienne droite. L'artériographie a objectivé trois dilatations anévrismales au niveau de l'artère communicante postérieure droite de 8,3mm, au niveau du segment M2 de la sylvienne droite de 4 mm (A) et enfin au niveau de la bifurcation sylvienne gauche de 4mm (B). La patiente a bénéficié d'un traitement endovasculaire des deux anévrismes à droite avec un rendez-vous de 3 mois pour sécuriser l'anévrisme gauche. La prévalence des anévrismes intracrâniens dans la population générale est évaluée de 1 à 5%. Seulement 15% de ces patients ont des anévrismes multiples, et 7% de ces patients ont plus de quatre anévrismes. La stratégie thérapeutique vise à sécuriser d'abord l'anévrisme qui a saigné et de traiter ultérieurement les autres anévrismes en fonction de leur taille et du risque hémorragique.

**Figure 1:**
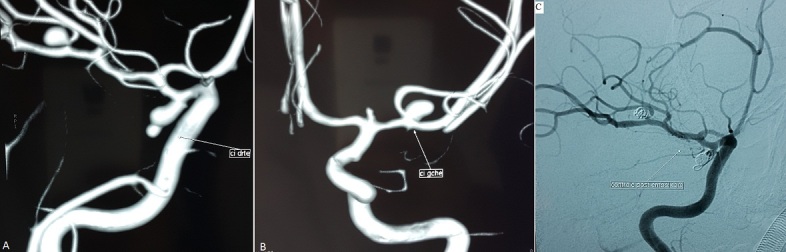
A) artériographie de la carotide interne droite montrant le sac anévrismal de la communicante postérieur mesurant 8.3 mm et le sac anévrismal du segment M2 de la sylvienne droite de 4 mm; B) artériographie de la carotide interne gauche montrant le sac anévrismal de la bifurcation sylvienne gauche de 4mm; C) contrôle artériographique montrant l'exclusion des deux anévrysmes à droite par des coïls

